# Emulating Quantum Entangled Biphoton Spectroscopy Using Classical Light Pulses

**DOI:** 10.1021/acs.jpclett.3c01714

**Published:** 2023-08-31

**Authors:** Liwen Ko, Robert L. Cook, K. Birgitta Whaley

**Affiliations:** † Department of Chemistry, University of California Berkeley, Berkeley, California 94720, United States; ‡ Kavli Energy Nanoscience Institute at Berkeley, Berkeley, California 94720, United States

## Abstract

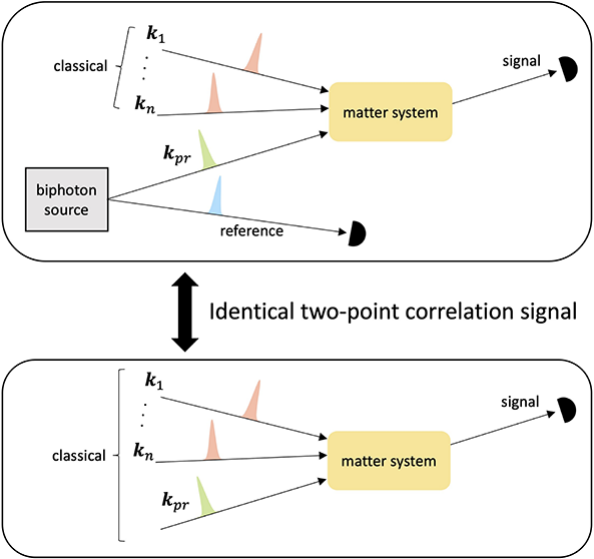

We show that for a class of quantum light spectroscopy (QLS) experiments using *n* = 0, 1, 2, ··· classical light pulses and an entangled photon pair (a biphoton state) where one photon acts as a reference without interacting with the matter sample, identical signals can be obtained by replacing the biphotons with classical-like coherent states of light, where these are defined explicitly in terms of the parameters of the biphoton states. An input-output formulation of quantum nonlinear spectroscopy is used to prove this equivalence. We demonstrate the equivalence numerically by comparing a classical pump–quantum probe experiment with the corresponding classical pump–classical probe experiment. This analysis shows that understanding the equivalence between entangled biphoton probes and carefully designed classical-like coherent state probes leads to quantum-inspired classical experiments that yield equivalent signals and provides insights for the future design of QLS experiments that could provide a true quantum advantage.

Spectroscopy using quantum light, in particular, using nonclassical pulses containing individual or entangled pairs of photons, has attracted much interest in recent years, both theoretically and experimentally, due to its potential to exploit the nonclassical properties of light to outperform classical spectroscopy.^1−16^ Quantum light spectroscopy (QLS) has been proposed to enable simplification of congested spectra,^4^ to target specific doubly excited states,^16^ to improve the signal-to-noise ratio of linear spectroscopy,^3^ and to measure dephasing rates with high temporal resolution.^6^ Understanding the extent to which such QLS experiments provide a quantum advantage requires careful comparison with experiments using the classical states of light. For example, the relationship between a quantum pump–quantum probe experiment and classical two-dimensional (2D) spectroscopy experiments is examined in ref (17).

In this paper, we show that for a certain class of QLS experiments, the use of entangled photon pairs can be replaced with coherent states of light, which behave classically when normal-ordered field correlations are evaluated (Figure 1). This is done in two steps. First, we show that for QLS experiments using entangled photon pairs with one photon being measured without interacting with the matter system,^2−4,7,8,13−15^ the entangled photon pair can be replaced with a specially designed single photon Fock state, since measuring one photon effectively collapses the other photon into a single photon state. Thus, two-photon entanglement offers no true quantum advantage in these QLS experiments. This has been pointed out previously by^18^ using quantum information theory arguments, and an analysis of the quantum information that one can extract from a two-level system using an entangled photon pair versus that extracted using a single photon Fock state has been provided in ref (19). In the context of single molecule biphoton spectroscopy that measures a photon in the longer time scale of fluorescence, ref (20) has recently shown that if all scattered photons can be measured, then the entanglement in the photon pair offers no advantage over a single photon. Nevertheless, there may still be practical advantages when such entangled photon pairs are used with one photon acting as a reference without interacting with matter. For example, pure single photon Fock states are more difficult to produce experimentally than entangled photon pairs,^21^ and a visible idler photon may be easier to detect than an infrared signal photon.^5^

**Figure 1:**
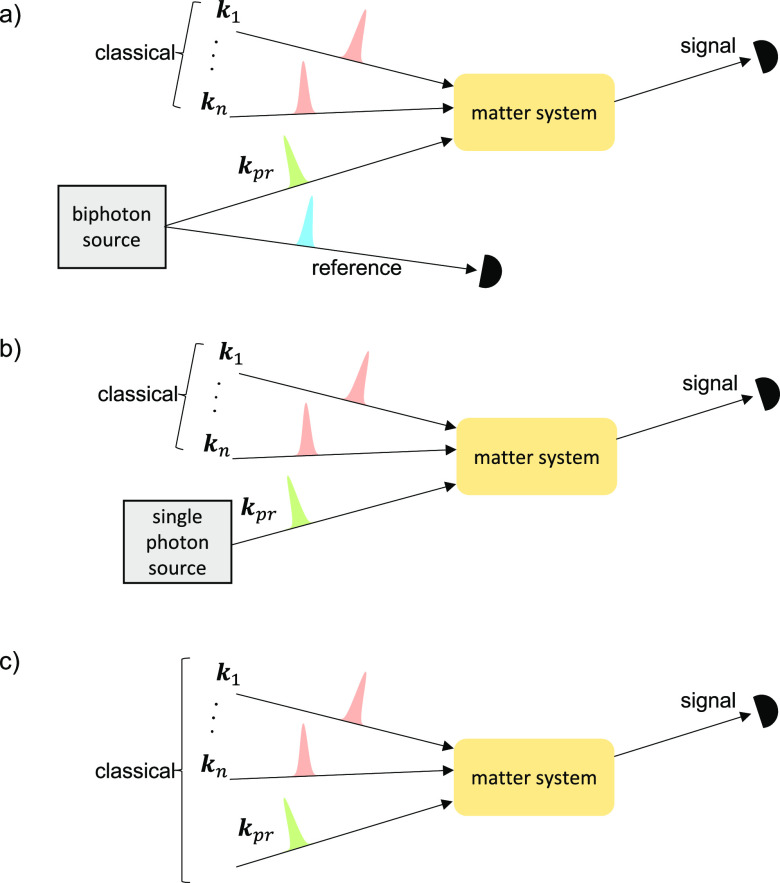
Spectroscopic schemes with *n* = 0, 1, 2, ··· classical pulses and (a) a biphoton probe pulse with one of the photons acting as a reference without interacting with matter, (b) a single photon Fock state probe pulse, and (c) a classical probe pulse containing one photon on average. The signal is measured in all cases in the direction of the probe field. The equivalence between schemes (a) and (b) is referred to as Equivalence 1 in the paper. The equivalence between schemes (b) and (c) is referred to as Equivalence 2 in the paper. Equivalence 2 holds under the conditions that (1) there is no phase matching of the *n* classical pulses into the direction of the probe field, and (2) the signal takes the form of a two-point correlation function (e.g., photon flux, frequency-resolved photon count, or *g*^(1)^ coherence function).

Second, and this is the main theoretical result of the paper, we show that for a class of experiments using *n* classical pulses (*n* = 0, 1, 2, ···) and a single photon Fock state probe pulse, identical signals can also be obtained using a coherent state pulse having the same temporal profile and containing one photon on average. Furthermore, if one uses a coherent state probe with the same temporal profile but containing many photons on average, then the signal can be amplified by a factor equal to the average number of photons. Taken together with the equivalence of biphoton and single Fock state probes, this means that the spectral features obtained from experiments with biphoton probe pulses can be exactly reproduced and also amplified by carefully designed coherent state probe pulses. Such quantum-inspired coherent state probes are much simpler to implement and thus far more preferable than biphoton states for experimental implementation.

We focus our analysis here on spectroscopy experiments for which the signal is measured in the direction of the probe. For the case of a single classical pump, *n* = 1, this allows direct comparison with conventional classical pump–probe spectroscopy and the entangled biphoton probe version of this that was proposed in ref (4). Other spectroscopy experiments where the signal is measured in directions other than the probe are not considered here but can be analyzed similarly using the method we present here. For the equivalence between a single photon Fock state and a single photon coherent state to hold, we require that the classical pulses are incident from different directions than the probe pulse and that there is no phase matching of the classical pulses into the direction of the probe pulse. In fact, the latter requirement includes the former as a special case. Neither of these are onerous requirements for experiments.

In this work, we restrict the signal detection to normal-ordered two-point correlation measurements that contain one creation operator and one annihilation operator in the transmitted probe field, for example, photon flux ⟨*a*^†^(*t*) *a*(*t*)⟩, frequency-resolved photon count ⟨*a*^†^(ω)*a*(ω)⟩, or the unnormalized *g*^(1)^ correlation function ⟨*a*^†^(*t*_2_)*a*(*t*_1_)⟩. We note that *g*^(1)^ is complex-valued and therefore not a quantum mechanical observable in the strict sense, but the real and imaginary parts of *g*^(1)^ can be measured separately using, for example, a Mach–Zehnder interferometer.^22^ The detection of higher-order coherence functions such as *g*^(2)^ (a four-point correlation function) is not considered here. The key reason for the equivalence between the Fock state probe and the coherent state probe in this class of experiments lies in the fact that they have the same two-point correlation function ⟨*a*^†^(*t*_2_)*a*(*t*_1_)⟩. While the one-point correlation functions ⟨*a*(*t*)⟩, ⟨*a*^†^(*t*)⟩, and other two-point correlation functions such as ⟨*a*(*t*_2_) *a*(*t*_1_)⟩ and ⟨*a*^†^(*t*_2_)*a*^†^(*t*_1_)⟩ are different for the two probes, their corresponding signals appear only under other phase matching conditions and do not appear in the direction of the probe field. So measuring the signal in this direction, as indicated in Figure 1, isolates signals that are dependent only on ⟨*a*^†^(*t*_2_)*a*(*t*_1_)⟩ and thereby ensures the desired equivalence. We note that this result is a generalization of our previous result^23^ that the output photon flux is the same under single photon Fock state and single photon coherent state excitation of a matter system in the ground state.

The matter system in this work consists of many molecules distributed over a volume of space, thus giving rise to the phase matching conditions. A single photon Fock state can generate entanglement between different uncoupled molecules, while a coherent state cannot generate such entanglement. Regardless of the difference in the collective matter state, we show here that the two-point correlation signals of the output light are the same. In a different context of a single molecule system, it has been shown^12,23^ that a single photon Fock state and a coherent state containing one photon on average give rise to the same excited state dynamics in the molecule. However, it is important to recognize that despite this similarity, there is also a difference in the overall dynamics of the reduced matter system, since the ground-excited state coherence is identically zero under a single photon Fock state excitation and nonzero under a coherent state excitation.^23^

Combining the equivalence between signals from biphotons and single photon Fock states and the equivalence between signals from single photon Fock states and single photon coherent states, we can then establish a class of QLS experiments that can be performed by using only classical light. We now begin the formal analysis.

## Equivalence 1: Equivalence between Signals from Biphoton and Single Photon Fock State Probes

Consider an experiment in which one probes a matter system using an entangled photon pair, whose density matrix is denoted as ρ_*AB*_. Photon A (e.g., the green pulse in Figure 1a) interacts with the system, and the resulting output optical field is measured subsequently. Photon B (e.g., the blue pulse in Figure 1b) is measured directly without interacting with anything. Note that in this section focused on the equivalence between signals from biphoton and single photon Fock state probes, we shall impose no restriction on the observables to be measured in each of the two photon fields. For each realization of the experiment, a joint measurement of both photon fields is recorded as (α, β) where α represents the signal in photon field A in that experimental realization, (e.g., whether or not a photon is present or the measured frequency of the photon), and β represents the measurement outcome of photon field B in the same experimental realization. Averaging over the signal α for fixed β with repeated measurements, one obtains the final reference-averaged signal as (*S*, β), where *S* is the averaged signal of photon field A. Each β corresponds to a value of the averaged signal *S*, so we shall henceforth abbreviate (*S*, β) as *S*_β_, representing the averaged signal of photon field A that is conditioned on the measurement outcome β of photon field B. It is sometimes suggested that the correlation between the pair of photons A and B enhances the signal *S*_β_.^4,8^ However, we show below that the conditional signal *S*_β_ can in fact be constructed alternatively using just single photon states that are parametrized by β. In other words, in the experimental scheme of Figure 1a, quantum entanglement between the two probe photons offers no fundamental advantage in learning about the matter system since exactly the same results can be obtained using just single photon states. This has also been pointed out by Stefanov in ref (18).

To derive the single photon state that produces the same signal, *S*_β_, we first note that measuring photon B projects the photon pair state to Π_β_ρ_*AB*_Π_β_, where Π_β_ is the projector onto the eigenspace of measurement outcome β. Since no further measurement is performed on photon B, photon A is then completely characterized by the reduced density matrix obtained by tracing the projected state over photon B:
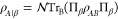
1Here  is a normalization factor to ensure unit trace. eq 1 tells us that measuring the reference photon field B with outcome β effectively collapses the input field of photon A into single photon state ρ_*A*|β_. Therefore, the conditional signal *S*_β_ can also be obtained exactly by probing the system with the single photon state ρ_*A*|β_.

As an example, consider the frequency-entangled photon pair
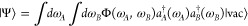
2where Φ(ω_*A*_,ω_*B*_) is the biphoton wave function,^22^*a*_*A*_(ω_*A*_) (*a*_*B*_(ω_*B*_)) is the bosonic annihilation operator of frequency mode ω_*A*_ (ω_*B*_) in photon field A (B), and |vac⟩ is the vacuum state of both fields. The operators *a*_*j*_(ω) satisfy the bosonic commutation relations: [*a*_*j*_(ω), *a*_*j*′_(ω′)] = [*a*_*j*_^†^(ω), *a*_*j*′_^†^(ω′)] = 0 and [*a*_*j*_(ω), *a*_*j*′_^†^(ω′)] = δ_*j*, *j*′_δ(ω – ω′). If we condition the experiment on measuring photon B at some reference frequency ω_*B*_ = ω_*r*_, then the corresponding projection operator Π_*ωr*_ is proportional to the outer product of the unnormalized state *a*_*B*_^†^(ω_*r*_)|vac⟩ and its adjoint, i.e.,
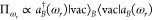
3where |vac⟩_*A*_ or |vac⟩_*B*_ denotes the vacuum state for the photon field A or B. The projected photon pair state becomes
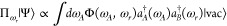
4which turns out to be a product state between the two photon fields A and B in this case. Therefore, the reduced state of photon field A, Tr_*B*_(Π_ω_*r*__|Ψ⟩⟨Ψ|Π_ω_*r*__), is a pure state, i.e.,
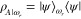
5with
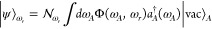
6where  is the normalization factor. Now the conditional signal can be alternatively obtained by using the single photon state of eq 6. Note that the frequency profile of this single photon state is explicitly given by evaluating the biphoton wave function Φ(ω_*A*_, ω_*B*_) at ω_*B*_ = ω_*r*_.

The equivalence between signals from biphoton and single photon Fock state probes can be understood in a slightly different way by considering the photon correlation functions. For example, if one is interested in some property of the photon field A, represented by the quantum operator *O*_*A*_, given that a photon with a frequency of ω_*r*_ is observed in the photon field B, one would typically need to evaluate the correlation function^4,8^
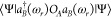
7Since , eq 7 is equal to the expectation value
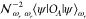
8with respect to the reduced single photon state |ψ⟩_ω_*r*__, up to a normalization constant  that can be determined from the biphoton wave function Φ(ω_*A*_, ω_*B*_).

## Equivalence 2: Equivalence between Signals from Single Photon Fock State and Single Photon Coherent State Probes

We now consider the class of experiments where *n* classical pump pulses (with wavevectors **k**_1_, ···, **k**_*n*_) and a single photon Fock state or a single photon coherent state probe pulse (with wavevector **k**_pr_), treated quantum mechanically, interact with a matter system. These are illustrated in Figure 1b and Figure 1c, for a single photon Fock state probe and a single photon coherent state probe, respectively. The classical pulses are incident in different directions from the quantum probe pulse, with the directions selected so that there is no phase matching of the classical pulses into the direction of the single photon probe. These conditions can be summarized as
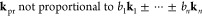
9where *b*_*i*_ = 0, 1, 2, ··· can be any non-negative integer, up to a reasonable number of orders of interaction. The case of *n* = 0 corresponds to the linear absorption of the single photon probe pulse; the case of *n* = 1 corresponds to a classical pump–single photon probe experiment. We place no restriction on the relative time ordering of the pulses. The signal is restricted to be normal-ordered two-point correlations that contain one creation operator and one annihilation operator in the probe field, e.g., photon flux ⟨*a*_pr,out_^†^(*t*) *a*_pr,out_(*t*)⟩, frequency-resolved photon count ⟨*a*_pr,out_^†^(ω) *a*_pr,out_(ω)⟩, or the *g*^(1)^ coherence function ⟨*a*_pr,out_^†^(*t*_2_) *a*_pr,out_(*t*_1_)⟩. We claim that the final signal coming from the single photon Fock state probe
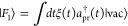
10is equal to the signal from a coherent state probe
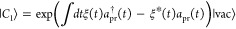
11having the same temporal profile ξ(*t*) and containing on average a single photon. The temporal profile ξ(*t*) is normalized according to ∫*dt* |ξ(*t*)|^2^ = 1. If the coherent state has *m* photons on average, i.e., the state
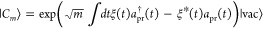
12then the probe field absorption and stimulated emission signal will be amplified by a factor of *m*.

The key to this equivalence between experiments carried out with Fock state probes and coherent state probes is that pulses from these two probes have the same two-point correlation function, ⟨*a*_pr_^†^(*t*_2_)*a*_pr_(*t*_1_)⟩. As already noted in the introduction, even though they have different two-point correlation functions ⟨*a*_pr_^†^(*t*_2_)*a*_pr_^†^(*t*_1_)⟩ and ⟨*a*_pr_(*t*_2_)*a*_pr_(*t*_1_)⟩ and different one-point correlation functions ⟨*a*_pr_^†^(*t*)⟩ and ⟨*a*_pr_(*t*)⟩, these other correlation functions do not contribute to the observed signal due to phase matching. Together with the explicit parametrization of the coherent state pulse in terms of the single photon frequency profile ξ(ω) = Φ(ω, ω_*r*_) obtained from the biphoton state in eq 6, this will allow replacement of a spectroscopic experiment using an entangled probe by experiments using a coherent state probe.

We now prove the equivalence explicitly by analyzing the signals using an input-output formulation of quantum nonlinear spectroscopy. This approach is based on a perturbative expansion of the signal observables in the Heisenberg picture, distinct from the more common approach of perturbing the combined system plus field density matrix in the interaction picture.^1,9^ The input-output formulation simplifies the theoretical analysis by focusing on the signal observables and using standard results from the input-output formalism of quantum optics.^23−25^

Our analysis will focus on the frequency-resolved photon count signal ⟨*a*_pr_^†^(ω)*a*_pr_(ω)⟩. The analysis for other two-point correlation signals, such as photon flux and *g*^(1)^ coherence function, follows almost identically. In the Heisenberg picture, the photon count of the transmitted probe at frequency ω is proportional to
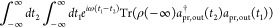
13Here ρ(−∞) is the initial combined system plus probe field state, assumed to be a product state between the matter system ρ_*M*_ and the field ρ_*F*_, and the trace operator is evaluated over both the matter and the field degrees of freedom. *a*_pr,out_(*t*) is the output field operator of the probe field. This output field operator is the result of time-evolving the input field operator in the Heisenberg picture with the combined matter and field Hamiltonian, and thus it mixes the field and matter degrees of freedom.^23^ The time domain field operator *a*(*t*) is related to the frequency domain field operator *a*(ω) by the Fourier relation
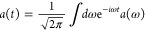
14Therefore, *a*(*t*) also satisfies the bosonic commutation relations.^22^

Although not necessary for the remaining derivation in this paper, we note that eq 13 is expressed in ref (4) in a different form in the interaction picture as
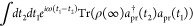
15where *a*_pr_(*t*) is now the input field operator of the probe field. ρ(∞) is the combined system plus field state in the interaction picture, evolved to a time longer than those of *t*_1_ and *t*_2_. The term ρ(∞) is somewhat nonintuitive. To show the equality between eqs 13 and 15, one considers how the input and output operators are related by unitary time-evolution operators. This is described in detail in Section A of the Supporting Information.

Under the dipole-electric field interaction and taking the zeroth-order Hamiltonian as the pure system plus pure field Hamiltonian, we can write the interaction picture Hamiltonian as
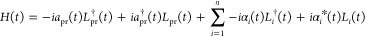
16eq 16 consists of the system interaction with the quantum probe field, represented by the field operator *a*_pr_(*t*), and with *n* other classical pulses, represented by their complex-valued coherent amplitudes α_*i*_(*t*). The operators *L*_pr_ and *L*_*i*_ are the matter deexcitation components of the dipole operator corresponding to the probe field and the field of the i-th classical pulse, respectively. In the interaction picture, *L*(*t*) = *e*^*iH*_sys_*t*^*Le*^–*iH*_sys_*t*^ is a purely system operator (setting ℏ = 1).

Under the Hamiltonian of eq 16, the input–output relation for the probe field is^23−25^
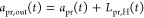
17with *a*_pr,out_(*t*) the output probe field operator and *a*_pr_(*t*) the input probe field operator. Here *L*_pr,H_(*t*) is the Heisenberg evolved operator, defined as *U*^†^(*t*) *L*_pr_(*t*) *U*(*t*), where *U*(*t*) is the time-evolution operator that solves the Schrodinger equation in the interaction picture, i.e., *dU*(*t*)/*dt* = −*iH*(*t*) *U*(*t*). The physical interpretation of eq 17 is that the output electric field is equal to the input electric field plus the electric field generated by the matter dipole moment. *L*_pr_(*t*), without the subscript “H”, will denote the operator in the interaction picture, which, as noted above, is a purely system operator. In contrast, *L*_pr,H_(*t*) now mixes the system and field degrees of freedom. Performing a perturbative expansion on the backward Heisenberg equation of motion for *L*_pr,H_(*t*),^26^ we have
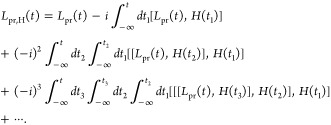
18The first term on right-hand side of eq 18 can be interpreted as the matter dipole moment without interacting with the light, the second term as the matter dipole moment due to interacting with the field once, the third term as the matter dipole moment due to two interactions with the field, and so on. After the commutators are expanded, each term becomes a product of a pure system operator and a pure field operator. Therefore, the expectation values of *L*_pr,H_(*t*) with respect to an initial product state can be readily evaluated.

Substituting eqs 17 and 18 into the Tr operator in eq 13, we obtain the following expansion for the two-point correlation function of the output signal:
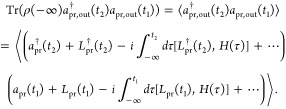
19Here we have adopted the conventional notation of using an angled bracket ⟨*Ô*⟩= Tr(ρ(−∞) *Ô*) to denote the expectation value of operator *Ô* with respect to the initial system plus field state ρ(−∞).

We now expand the right-hand side of eq 19 on the order of *L*_pr_. To show that *L*_pr_ is indeed proportional to a small parameter, first notice that *L*_pr_, *a*_pr_, and the *L*_i_ and α_*i*_ from the semiclassical terms of the Hamiltonian (eq 16) all have the same dimension of  (setting ℏ = 1). Since ⟨*L*_pr_^†^(*t*)*L*_pr_(*t*)⟩ (⟨*L*_*i*_^†^(*t*)*L*_*i*_(*t*)⟩) is at most equal to the spontaneous emission rate into the probe field (*i*^th^ classical field), where the maximum rate is obtained when the matter state is in the bright state of the corresponding field mode, we assign an order of magnitude value
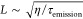
20to each *L*, where τ_emission_ is the time scale of spontaneous emission into the polarization of that field mode, and η is the geometric factor of the field mode.^23,27^ η is less than 1 because a paraxial mode in an experiment usually covers only a small fraction of all light with the polarization of that paraxial mode. For a light pulse containing an average of *m* photons, we have ∫*dt* ⟨*a*^†^(*t*)*a*(*t*)⟩ = *m* (for classical pulses, we replace operator *a*(*t*) with coherent amplitude α(*t*)). Therefore, we assign an order of magnitude value
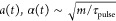
21to each *a*(*t*) or α(*t*), where τ_pulse_ characterizes the pulse duration. In typical visible spectroscopy experiments with atomic and molecular samples, τ_emission_ ≫ τ_pulse_, the matter system dynamics is observable before spontaneous emission removes the excitation. Furthermore, since *m* ≫ 1 for typical classical pulses and *m* = 1 for the single photon probe pulse, we conclude that the magnitude of *L* is much smaller than the magnitudes of *a*_pr_ and α_*i*_, justifying an expansion in powers of the *L* operators. We then choose to expand eq 19 only in orders of *L*_pr_, since the orders of *L*_*i*_ do not affect the main result, i.e., the equivalence of signals originating from a single photon Fock state probe and a single photon coherent state probe. Furthermore, since *L*_*i*_ always appears together with the classical pulse amplitude α_*i*_, the effect of *L*_*i*_ is amplified by a factor of , so *L*_pr_ becomes indeed the smallest parameter in the expansion of eq 19. We now analyze the three lowest-order contributions to the expansion.

### Zeroth-Order Term (∼ *L*_pr_^0^)

The only zeroth-order term in eq 19 is ⟨*a*_pr_^†^(*t*_2_)*a*_pr_(*t*_1_)⟩, the transmitted probe without any interaction with matter. This expectation value is the same for both the single photon Fock state (eq 10) and the single photon coherent state (eq 11), namely
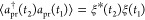
22where ξ(*t*) is the pulse shape. For the *m*-photon coherent state (eq 12), eq 22 is amplified by a factor of *m*.

### First-Order Terms (∼*L*_*pr*_^1^)

Any first-order term in eq 19 must be the expectation value of a product between *a*_pr_^(†)^ and another term containing a single factor of *L*_pr_, or its complex conjugate. In other words, only the semiclassical part of the Hamiltonian can contribute in the commutators of eq 19; otherwise, there will be more than one factor of *L*_pr_. Specifically, the first-order terms take the form
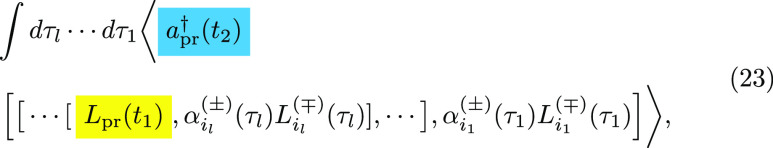
23and its complex conjugates. Here *l* = 0, 1, 2,···, and each *i* index can denote any one of the *n* classical field interactions. The probe field operators (i.e., *a*_pr_ or *a*_pr_^†^) are highlighted in blue, while the matter operators associated with the probe field (i.e., *L*_pr_ or *L*_pr_^†^) are highlighted in yellow. When *l* = 0, eq 23 reduces to ⟨*a*_pr_^†^(*t*_2_)*L*_pr_(*t*_1_)⟩. The notation α_*i*_^(±)^(τ) *L*_*i*_^(∓)^(τ) means either α_i_^*^(τ) *L*_*i*_(τ) or α_*i*_(τ) *L*_*i*_^†^(τ).

We note that the optical signal expression of eq 23 applies not just to the case of a single molecule but also to the case of many molecules. This is because the matter operators *L* of different molecules commute with each other, so that eq 23 is nonzero only if all of the *L* operators in the commutator originate from the same molecule. This argument applies to all signal expressions in the remainder of the paper. The fact that there are many molecules in our matter system gives rise to phase matching conditions, which we describe in the following paragraph.

Physically, eq 23 represents the heterodyne measurement between the probe field (i.e., the *a*_pr_^†^ in the first line) and the field generated by the matter polarization that is induced by the interactions with the classical fields (the second line). In all of the first-order terms, the probe field expectation value factored out as ⟨*a*_pr_^†^(*t*)⟩ or ⟨*a*_pr_(*t*)⟩. These one-point correlation functions are zero for Fock state inputs and nonzero for coherent state inputs; therefore, eq 23 is different for the Fock state and coherent state inputs. However, the optical signal generated by the matter polarization has the phase matching condition^26,28,29^
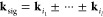
24where the *k*_*i*_ on the right-hand side are the wavevectors of the classical pulses. This means that **k**_sig_ must be in a different direction than the probe field direction **k**_pr_, due to our assumption of the beam geometry in eq 9, i.e., the probe pulse is not phase matched with any of the classical pulses. Therefore, the probe field signal of eq 23 will vanish, because it is not phase-matched to the matter polarization. At the molecular level, this means that in our beam geometry, the polarization from different molecules will generate destructively interfering signals and result in a zero overall signal. Hence the first-order (∼*L*_pr_^1^) signal does not contribute to the probe field output.

### Second-Order Terms (∼*L*_pr_^2^)

There are two types of second-order terms. The first type is related to spontaneous emission and takes the form
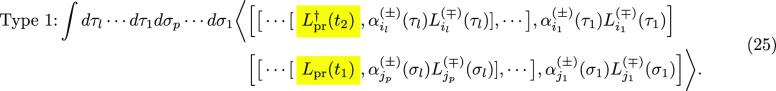
25The integrand is a product of two nested commutators. Here *l* and *p* can take values of 0, 1, 2, ···. Each of the *i* and *j* indices can be any one of the *n* semiclassical interactions of the Hamiltonian. We take only the semiclassical part of the Hamiltonian in the commutators, since there is already one *L*_pr_ in each of the two nested commutators. Otherwise eq 25 will contain more than two *L*_pr_, becoming a higher-order term. In the case of *l* = *p* = 0, eq 25 becomes ⟨*L*_pr_^†^(*t*_2_) *L*_pr_(*t*_1_)⟩: this represents spontaneous emission into the probe field without any interaction with the classical pulses. Since eq 25 contains no probe field operator (i.e., no *a*_pr_ or *a*_pr_^†^ terms), the expectation value is the same for all input field states, regardless of the phase matching conditions. For completeness, we note that the phase matching condition for these terms is^26^
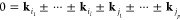
26The second type of second-order terms is related to absorption and stimulated emission, and has the form of
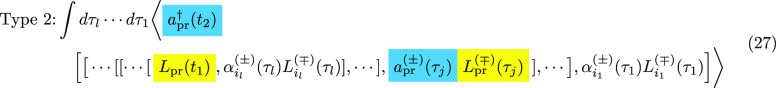
27and its complex conjugate. In the nested commutator expression, there is exactly one interaction with the quantum probe field. Physically, eq 27 represents the heterodyne measurement between the probe field (i.e., the *a*_pr_^†^ in the first line) and the field generated by the matter polarization that is induced by one interaction with the quantum probe field and a number of interactions with the classical fields (the second line). The notation of the probe interaction term *a*_pr_^(±)^(τ_*j*_) *L*_pr_^(∓)^(τ_*j*_) stands for either product *a*_pr_^†^(τ_*j*_) *L*_pr_(τ_*j*_) or *a*_pr_(τ_*j*_) *L*_pr_^†^(τ_*j*_).

When the probe field interaction is *a*_pr_^†^(τ_*j*_)*L*_pr_(τ_*j*_), the probe field correlation in eq 27 factorizes out as ⟨*a*_pr_^†^(*t*_2_) *a*_pr_^†^(τ_*j*_)⟩, which is zero for Fock state inputs and nonzero for coherent state inputs. Now the optical signal generated by the matter polarization has the phase matching condition of
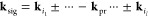
28where the right-hand side contains only one probe field wavevector *k*_pr_, and all other *k*_*i*_ are the classical pulse wavevectors. But as discussed above, **k**_sig_ cannot be in the same direction as **k**_pr_ due to our assumption of the beam geometry in eq 9. Therefore, the final signal is not phase matched in the probe field direction **k**_pr_ and will vanish. So neither a Fock state input nor a coherent state input will produce any signal from the *a*_pr_^†^(τ_*j*_)*L*_pr_(τ_*j*_) interaction in this direction.

On the other hand, when the probe field interaction in eq 27 is *a*_pr_(τ_*j*_) *L*_pr_^†^(τ_*j*_), the field correlation now factorizes as ⟨*a*_pr_^†^(*t*_2_)*a*_pr_(τ_*j*_)⟩, which is the same for both the single-photon Fock state and single-photon coherent state inputs, regardless of the phase-matching condition. These terms represent the transient absorption and/or stimulated emission of the probe field due to the interaction with the classical pulses. In this case the phase matching condition of the optical signal generated by the matter polarization is now
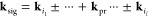
29where the right-hand side consists of only one probe field wavevector *k*_pr_, and all other *k*_*i*_ are the classical pulse wavevectors. We see that now if the classical pulse wavevectors cancel each other out pairwise, then we will have the correct phase matching condition of *k*_sig_ = *k*_pr_ that results in a nonzero final signal in the probe field.

Due to the weak nature of the interaction between a single photon and a molecule (for example the probability for a chlorophyll molecule to absorb a single photon is at most on the order of ∼10^–6^ due to phonon dephasing^23,30^), it is reasonable to truncate eq 19 up to second order in *L*_pr_. This second-order truncation corresponds to one interaction with the probe field in the language of classical nonlinear spectroscopy.^28^

We may then combine the analysis for all of the terms up to second order in *L*_pr_ (i.e., eqs 22, 23, and 25), and the two cases in eq 27). Doing this, we see first that while the first-order contribution eq 23 and the first case of the second type of second-order contribution eq 27 yield different values for Fock state and coherent state inputs, neither of these terms appears in the final signal due to the phase matching constraint; therefore, they cannot contribute to a difference between Fock and coherent state inputs. In contrast, the zeroth-order contribution eqs 22, the first type of the second-order contribution (25), and the second case of the second type of second-order contribution eq 27 yield the same value for both Fock state and coherent state inputs, and these terms do have the correct phase matching condition to contribute to the final signal. Therefore, provided that the coherent state has the same temporal profile as the Fock state, a single photon Fock state probe and a single photon coherent state probe will produce exactly the same signal in the experimental setups of Figure 1b and 1c. Furthermore, a many photon coherent state probe with the same temporal profile will amplify the signals of eq 22 and the second case of eq 27 by a factor of *m*, where *m* is the average number of photons.

## Example: Pump Quantum-Inspired Probe (PQIP) Spectroscopy

**Figure 2:**
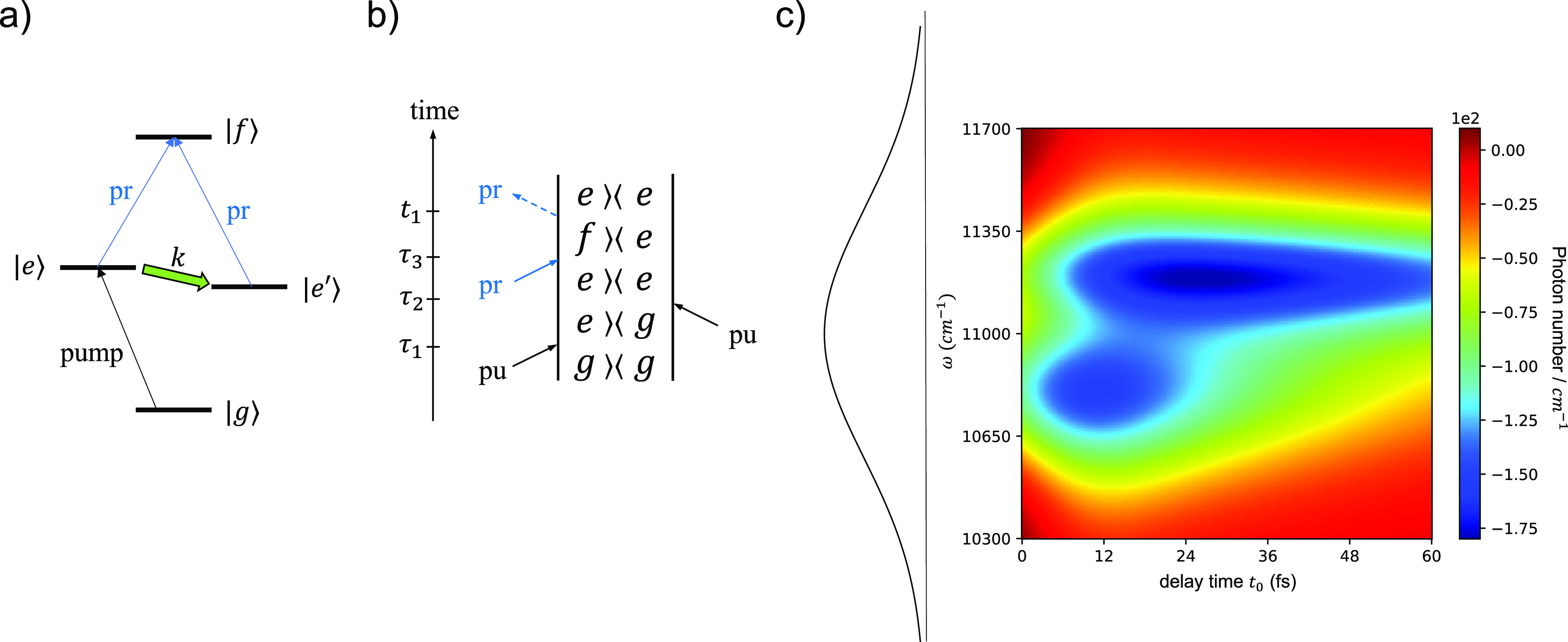
(a) Energy level scheme with four levels from ref (4), which we use for our numerical example. The pump pulse is resonant to only the |*g*⟩ → |*e*⟩ transition. The probe pulse is resonant to only the |*e*⟩ → |*f*⟩ and |*e*′⟩ → |*f*⟩ transitions. (b) Double-sided Feynman diagram representing the excited state absorption of the pump–probe signal. The order of the first two pump interactions can be switched. (c) Transient absorption spectrum due to a conventional classical probe. The spectrum plots the change in the probe field frequency-resolved photon count ⟨*a*_pr_^†^(ω)*a*_pr_(ω)⟩ at frequency ω, i.e., the signal photon number spectral density. This conventional classical probe has a Gaussian frequency profile *E*(ω) ∝ *e*^–(ω–ω_0_)^2^/2σ^2^^ (ω_0_ = 11000 cm^–1^, σ = 600 cm^–1^) and contains on average 10^6^ photons. The frequency distribution |*E*(ω)|^2^ of the input probe pulse is plotted to the left of the spectrum.

To demonstrate this equivalence between an entangled photon probe and a coherent state probe, we consider here the specific example of the classical pump - quantum probe experiment described theoretically in,^4^ which corresponds to the case of a single classical pump pulse, i.e., *n* = 1 in Figure 1. We then compare this experiment to the corresponding classical pump - quantum-inspired classical probe experiment, which we shall refer to as “pump quantum-inspired probe” (PQIP). In this experiment, a delta-function classical pump first excites a four-level matter system from the ground state |*g*⟩ to the singly excited state |*e*⟩, which transfers the excitation to another lower-energy singly excited state |*e*′⟩ irreversibly with a rate *k* (Figure 2a). These energy transfer dynamics are monitored by the transient absorption of a probe pulse (delayed by time *t*_0_ from the pump pulse) that excites |*e*⟩ or |*e*′⟩ into the doubly excited state |*f*⟩. In,^4^ the probe pulse was taken to be either a classical pulse or an entangled photon pair. Figure 2c shows the calculated transient absorption spectrum using a conventional classical probe pulse consisting of a single Gaussian with frequency width σ = 600 cm^–1^, covering both transition frequencies from |*e*⟩ and |*e*′⟩ to |*f*⟩. The structure of the two peaks centered at different delay times reveal the energy transfer dynamics from |*e*⟩ to |*e*′⟩.

In the case of a biphoton probe, the photon pair state |Ψ⟩ is given by eq 2, with the biphoton wave function Φ(ω_pr_, ω_*r*_). One photon (the reference photon) of the probe photon pair does not interact with the matter system, and its frequency ω_*r*_ is measured. The other photon (the probe signal photon) interacts with the matter system and is frequency-resolved. For each ω_*r*_, there is a transient absorption spectrum as a function of the signal frequency, ω and delay time *t*_0_. As discussed in ref (4), due to the frequency correlation in the entangled photon pair, by selecting different values of ω_*r*_, one can target specific frequency windows of the transient absorption spectrum, thereby simplifying the spectrum.

The theoretical analysis of these spectra obtained from biphoton pulses proceeds as follows. The pump–probe signal for a fixed reference photon frequency ω_*r*_ is the difference between the output and the input signals
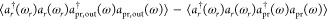
30If no reference photon is used, the pump–probe signal becomes
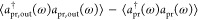
31Applying eqs 17 and 18, the lowest order term of eq 30, represented by the double-sided Feynman diagram of Figure 2b, is
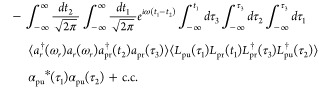
32Note that eq 32 originates from the second-order (∼*L*_pr_^2^) expansion term of the form of eq 27. Substituting in the delta-function classical pump α_pu_(*t*) ∝ δ(*t*), eq 32 is now proportional to
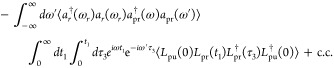
33Since the field correlation function in eq 33 with a time delay of *t*_0_ evaluates to
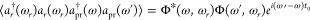
34The signal eq 33 can be expressed compactly as^4^
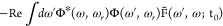
35where
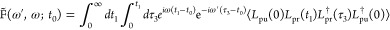
36is the frequency-domain matter correlation function defined in ref (4).

The detailed model of the matter system, the corresponding analytical form of *F̃*(ω′, ω; *t*_0_), and the analytical form of Φ(ω_pr_, ω_*r*_) are discussed in ref (4) and summarized in Section B of the Supporting Information. Similarly, if a single photon Fock state or a coherent state is used as the probe, then the pump–probe signal eq 31 becomes
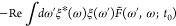
37where ξ(ω) is the frequency profile of the probe pulse (see eqs 10–12).

Comparing eq 35 and eq 37, we observe that if we choose a quantum-inspired coherent state probe with coherent amplitude ξ_pr_(ω) = Φ(ω, ω_*r*_), the final signal is exactly proportional to eq 35 at a fixed reference photon frequency ω_*r*_. Therefore, the classical pump–quantum probe experiment can be exactly reproduced using a standard classical pump–classical probe setup, with the only additional feature of requiring a pulse shaper for the quantum-inspired classical probe pulse. The shape of the quantum-inspired classical probe is parametrized by ω_*r*_, together with the other parameters of the biphoton pulse (see Supporting Information, Section B).

**Figure 3:**
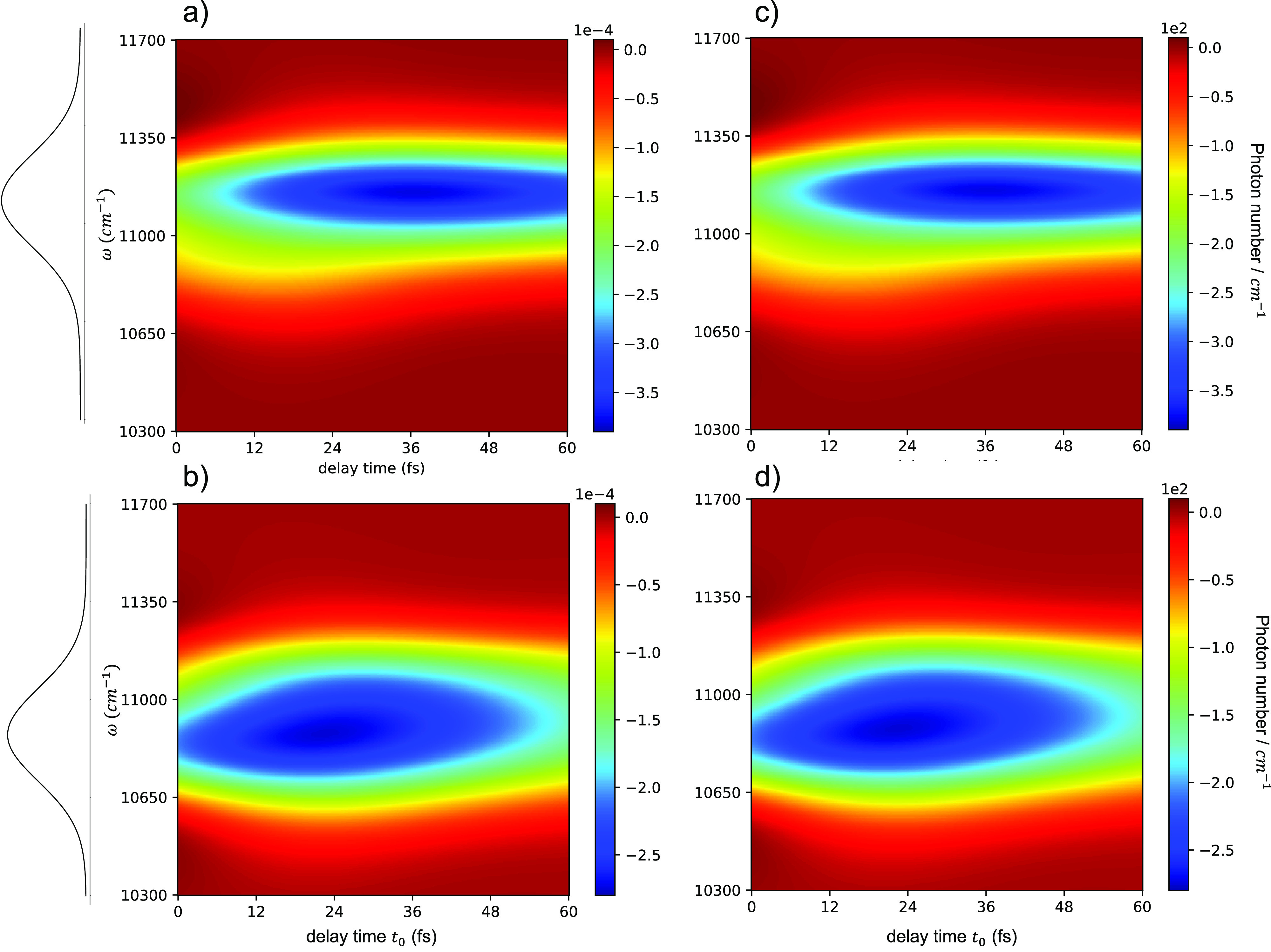
Transient absorption spectra obtained using (a,b) an entangled biphoton probe or (c,d) quantum-inspired classical probes. The signal is the change of the probe field frequency-resolved photon count ⟨*a*^†^(ω)*a*(ω)⟩ at frequency ω, i.e., the signal photon number spectral density. In panels (a) and (b), the signal is conditioned on the reference photon frequencies of (a) ω_*r*_ = 11400 cm^–1^ and (b) ω_*r*_ = 10400 cm^–1^. On the left of each spectrum is the frequency distribution |Φ(ω, ω_*r*_)|^2^ of the probe single photon for fixed ω_*r*_. In panels (c) and (d), classical probes with frequency profiles ξ(ω) = Φ(ω, ω_*r*_) are used, where ω_*r*_ = 11400 cm^–1^ in (c) and ω_*r*_ = 10400 cm^–1^ in (d), corresponding to panels (a) and (b), respectively. The classical probe pulses contain 10^6^ photons on average, resulting in 10^6^ times signal amplification. Note that the scales of the color bars in parts c and (d) are 10^6^ larger than those in parts a and (b).

The classical pump–quantum probe spectra with biphoton pulses, characterized by two choices of ω_*r*_, are shown in the left-hand panels (a) and (b) of Figure 3. The signal is detected in the probe beam direction, in accordance with the phase matching requirement discussed in Equivalence 2. The simplification of the spectra relative to the conventional pump–probe spectrum in Figure 2 is immediately evident, with the two peaks now clearly resolved, permitting a more detailed analysis of the coupled dynamics underlying the two spectra.

The corresponding PQIP spectra are shown in the right-hand panels (c) and (d) of Figure 3. In the numerical simulation, we use a classical probe containing an average of *m* = 10^6^ photons to amplify the final signal by a factor of 10^6^. As noted above, this has the additional benefit of making signal detection much easier experimentally than when using an entangled biphoton probe. When the amplified signals are normalized to the same reference value as that for panels (a) and (b), the left- and right-hand panels of Figure 3 are identical to within numerical precision, validating the PQIP analysis. The specific quantum-inspired pulses that produce the same spectra as the biphoton probe with ω_*r*_ values in panels (a) and (b) of Figure 2 are given explicitly in Section B of the Supporting Information.

## Conclusion

We have shown that for a class of QLS experiments consisting of *n* = 0, 1, 2··· classical pulses and an entangled photon pair probe in the scheme of Figure 1a), the use of the entangled photon pair can be replaced with a specially designed coherent state pulse, which behaves as classical light when normal-ordered field correlations are evaluated. The two main requirements for this equivalence to hold are the following: (1) there is no phase matching of the classical pulses into the direction of the probe field and (2) signal measurement takes the form of (time-integrated) photon flux, frequency-resolved photon count, or *g*^(1)^(*t*) correlation function.

The class of experiments described in this paper is a subset of QLS experiments, where the use of entangled photon pairs can be replaced with classical pulses. Whenever a biphoton input is used and a noninteracting reference photon *r* is measured at frequency ω_*r*_, so that the signal consists of field correlation functions of the form ⟨*a*_*r*_^†^(ω_*r*_) *a*_*r*_(ω_*r*_)*a*^†^(*t*_2_)*a*(*t*_1_)⟩, we showed that the signal can be reproduced with coherent state pulses that are specifically designed for a given biphoton state and reference frequency. In this work, we also demonstrated the validity of the analysis by explicit calculations of the signal for a classical pump–entangled photon probe experiment, showing numerical equivalence with the signal obtained from a classical pump with a coherent state pulse that is constructed according to the two equivalences.

Going beyond the scope of analysis in this paper, one may also consider the effect of the environmental background photon noise (i.e., dark counts) on spectroscopy with entangled photons.^3^ Here the signal-to-dark-count improvement offered by entangled photon pairs described in ref (3) can be achieved by using pulsed classical light in only the signal arm and detecting the output photon only in the time window Δ*t* that is set to be equal to the pulse duration. This is because the number of dark count photons during the detection time window is linearly proportional to Δ*t*, so by using a classical pulse with a short duration (i.e., small Δ*t*), one can reduce the number of dark count photons and increase the signal-to-dark-count ratio.

We can also consider the effect of noise due to the uncertainty of the photon number or the noise due to detector inefficiency. For simplicity, we shall refer here to both of these uncertainties as measurement shot noise. Using a 100% efficient photon detector, the detection of a single photon has zero shot noise. Therefore, the signal-to-noise ratio of a single photon from one-half of an entangled photon pair cannot be achieved using classical pulses. However, if the photon detector is not perfectly efficient, the detection of a single photon will contain a nonzero shot noise. Then the signal-to-noise ratio for the detection of a single photon can be achieved or surpassed using a coherent state pulse with large enough amplitude. This is because the signal-to-noise ratio of a coherent state under both perfectly efficient or inefficient photon detectors is equal to .^22^ Therefore, by increasing the coherent state amplitude, one can systematically improve the signal-to-noise ratio.

If we go beyond the dipole-electric field interaction and allowing for Raman scattering interactions, one can also show that the intensity correlated Raman signal in^8^ and the (1,0) component of the interferometric stimulated Raman signal in ref (2) can also be reproduced by classical pulses parametrized by the biphoton wave function and the reference photon frequency, since these signals depend on the same field correlation function as in eq 32.

Finally, we note that while some QLS experiments can be reproduced using carefully designed classical light sources as shown here, at the same time, the technologies for generation and detection of quantum light are maturing, raising the possibility of a new generation of QLS experiments. The equivalence between entangled biphoton probes and classical-like coherent state probes shown in this work leads to a new category of quantum-inspired classical spectroscopy experiments, such as the pump quantum-inspired probe experiment. An understanding of the range of applicability of the equivalence demonstrated here will provide insights for the future design of more powerful QLS experiments that cannot be replicated by suitably designed quantum-inspired classical pulses and that could provide a true quantum advantage for the study of electronic dynamics in complex systems.

## Supporting Information Available

Section A: Relationship between eqs 13 and 15. Section B: Numerical parameters for simulating the pump quantum-inspired probe spectra (PDF)
