# Supplementary material for: Emulating Quantum Entangled Biphoton Spectroscopy Using Classical Light Pulses
Source: J Phys Chem Lett. 2023 Aug 31;14(36):8050–9. doi: 10.1021/acs.jpclett.3c01714 (PMC10510434; doi:10.1021/acs.jpclett.3c01714)
Supplement: Supplementary file 1 — jz3c01714_si_001.pdf [file jz3c01714_si_001.pdf]

Supporting Information
for
Emulating Quantum Entangled Biphoton Spectroscopy Using Classical Light Pulses

Liwen Ko[*1,2], Robert L. Cook[1,2], and K. Birgitta Whaley[1,2]

[1] Department of Chemistry, University of California, Berkeley, CA 94720, USA
[2] Kavli Energy Nanoscience Institute at Berkeley, Berkeley, CA 94720, USA
[*] liwen.jko@berkeley.edu

A Relationship between Eqs. (13) and (15)

To show that the Heisenberg picture signal of Eq. (13) is equal to the interaction picture signal of Eq. (15), it suffices to show

$$\mathrm{Tr}\big(\rho(-\infty)a^\dagger_{\mathrm{pr,\ out}}(t_2)a_{\mathrm{pr,\ out}}(t_1)\big) = \mathrm{Tr}\big(\rho(\infty)a^\dagger_{\mathrm{pr}}(t_2)a_{\mathrm{pr}}(t_1)\big). \tag{A.1}$$

The presence of $\rho(\infty)$ in the interaction picture is not very intuitive, but this can be understood if we consider the following relation [1]:

$$a_{\mathrm{pr,out}}(t') = U^\dagger(t)a_{\mathrm{pr}}(t')U(t), \tag{A.2}$$

where $t > t'$. $U(t)$ is defined below Eq. (17). Taking a common time variable t, such that $t > t_1, t_2$. The left-hand side of Eq. (A.1) becomes

$$\mathrm{Tr}\big(\rho(-\infty)U^\dagger(t)a^\dagger_{\mathrm{pr}}(t_2)U(t)U^\dagger(t)a_{\mathrm{pr}}(t_1)U(t)\big). \tag{A.3}$$

Using the invariance of the trace under cyclic permutation and the unitary property $U(t)U^\dagger(t) = 1$, (A.3) becomes

$$\mathrm{Tr}\big(U(t)\rho(-\infty)U^\dagger(t)a^\dagger_{\mathrm{pr}}(t_2)a_{\mathrm{pr}}(t_1)\big). \tag{A.4}$$

Finally, taking $t \to \infty$, so that $U(t)\rho(-\infty)U^\dagger(t) \to \rho(\infty)$, we obtain the right-hand side of Eq. (A.1).

B Numerical parameters of Sec. 4

Using the two-state jump model in [2] for the matter system and following the notation in that work, we take $\omega_{fe} = 11000\,\mathrm{cm}^{-1}$, $\delta = 200\,\mathrm{cm}^{-1}$, $k = 120\,\mathrm{cm}^{-1}$, and $\gamma = 100\,\mathrm{cm}^{-1}$. From Eq. (19) of [2], we derive the matter correlation function

$$\begin{aligned}\tilde{F}(\omega', \omega; t_0) = e^{-i(\omega-\omega')t_0}\Big(&\frac{1}{(\omega-\omega'+i\gamma)}\frac{1}{(\omega-\omega_++2i\gamma)}\\ &+\frac{2i\delta}{k+2i\delta}\frac{1}{(\omega-\omega'+i(k+\gamma))}\frac{1}{(\omega-\omega_-+i(k+2\gamma))}\\ &-\frac{2i\delta}{k+2i\delta}\frac{1}{(\omega-\omega'+i(k+\gamma))}\frac{1}{(\omega-\omega_++2i\gamma)}\Big),\end{aligned} \tag{B.1}$$

where $\omega_\pm = \omega_{fe} \pm \delta$. We note that this is slightly different from Eq. (20) of [2]. We then multiply $\tilde{F}(\omega', \omega; t_0)$ by a factor of 20, so that around 10% of the probe is absorbed at the peak of the pump-probe spectrum.

The factor of 20 effectively takes into account the light beam geometry, the molecular dipole strength, and the number of molecules in the sample.

The biphoton wavefunction of [2] takes the Gaussian form

$$\Phi(\omega, \omega_r) = \mathcal{N} e^{-\frac{(\omega+\omega_r-\omega_0)^2}{2\sigma^2}} e^{-\beta[(\omega-\omega_0/2)T_2+(\omega_r-\omega_0/2)T_1]^2}, \tag{B.2}$$

where $\mathcal{N}$ is a normalization factor ensuring $\int d\omega d\omega_r |\Phi(\omega, \omega_r)|^2 = 1$, $\beta = 0.04822$, $\omega_0 = 22000\,\text{cm}^{-1}$, $\sigma = 1000\,\text{cm}^{-1}$, $T_1 = -19.69\,\text{fs}$, and $T_2 = 70.31\,\text{fs}$. If we choose a fixed value of ω_r, then the bivariate Gaussian biphoton wavefunction reduces to a single-variable Gaussian function $\propto e^{-(\omega-\omega_0')^2/2\sigma'^2}$ with a modified center frequency

$$\omega_0' = \left(\frac{1}{\sigma^2} + 2\gamma T_2^2\right)^{-1} \left[\frac{\omega_0 - \omega_r}{\sigma^2} + 2\gamma T_2\left(\frac{\omega_0}{2}(T_1 + T_2) - \omega_r T_1\right)\right] \tag{B.3}$$

and variance

$$\sigma' = \left(\frac{1}{\sigma^2} + 2\gamma T_2^2\right)^{-1/2}. \tag{B.4}$$

This gives the explicit form of the quantum-inspired classical probe pulse corresponding to the biphoton pulse, which is thus seen to depend on the biphoton parameters γ, T_1, T_2, ω_0 and σ, in addition to ω_r. When $\omega_r = 10400\,\text{cm}^{-1}$, the quantum-inspired pulse has $\omega_0' = 10874.81\,\text{cm}^{-1}$ and $\sigma' = 236.09\,\text{cm}^{-1}$. When $\omega_r = 11400\,\text{cm}^{-1}$, the quantum-inspired pulse has $\omega_0' = 11083.46\,\text{cm}^{-1}$ and $\sigma' = 236.09\,\text{cm}^{-1}$.

References

[1] Liwen Ko, Robert L. Cook, and K. Birgitta Whaley. Dynamics of photosynthetic light harvesting systems interacting with n-photon fock states. *J. Chem. Phys.*, 156(24):244108, 2022.

[2] F. Schlawin, K. E. Dorfman, and S. Mukamel. Pump-probe spectroscopy using quantum light with two-photon coincidence detection. *Phys. Rev. A*, 93:023807, Feb 2016.
